# The effect of selected rest break activities on reaction time, balance, and perceived discomfort after one hour of simulated occupational whole-body vibration exposure in healthy adults

**DOI:** 10.1080/07853890.2023.2244965

**Published:** 2023-08-12

**Authors:** Wadena D. Burnett, Michael Tweten, Udoka Okpalauwaekwe, Catherine Trask, Stephan Milosavljevic

**Affiliations:** a School of Rehabilitation Science, University of Saskatchewan, Saskatoon, SK, Canada; b College of Medicine, University of Saskatchewan, Saskatoon, SK, Canada; c Division of Ergonomics, KTH Royal Institute of Technology, Stockholm, Sweden

**Keywords:** Whole-body vibration, occupational exposure, accident prevention, ergonomics, laboratory simulation, reaction time

## Abstract

**Background & Objective***:* Negative health effects from occupational whole-body vibration (WBV) exposure during machinery operation include alterations in proprioception, vestibular function, reaction time, stress, motor response, and decrements in musculoskeletal health. To reduce WBV exposure during machinery operation, it may be possible to incorporate short rest break activities throughout the day. This study aims to determine if there are intervention activities that can minimize decrements in cognitive, proprioceptive, and musculoskeletal effects related to WBV exposure during machine operation.

**Materials & Methods:**

Eleven healthy adults participated in four 1-hour sessions of ecologically valid WBV exposure followed by one of four 5-minute activities: sitting, walking, 2 min of gaze stabilization exercise (GSE) coupled with 3 min of trunk mobility exercise (GSE + MOBIL), or 2 min of GSE coupled with a 3-minute walk (GSE + WALK). Baseline and post-activity measurements (rating of perceived discomfort, balance and postural sway measurements, 5-minute psychomotor vigilance task test) were submitted to a paired t-test to determine the effect of WBV exposure and activities on physical, cognitive, and sensorimotor systems and to a repeated measures ANOVA to determine any differences across activities.

**Results:**

We observed degradation of the slowest 10% reaction speed outcomes between baseline and post-activity after walking (7.3%, *p* < 0.05) and sitting (8.6%, *p* < 0.05) but not after GSE + MOBIL or GSE + WALK activities. Slowest 10% reaction speed after GSE + MOBIL activity was faster than all other activities. The rating of perceived discomfort was higher after SIT and WALK activities. There were no notable differences in balance outcomes.

**Conclusion:**

When compared to sitting for 5 min, an activity including GSE and an active component, such as walking or trunk mobility exercises, resulted in maintenance of reaction time after WBV exposure. If confirmed in occupational environments, GSE may provide a simple, rapid, effective, and inexpensive means to protect against decrements in reaction time after WBV exposure.

## KEY MESSAGES

1. A 5-minute intervention activity after 1 hour of occupational whole-body vibration (WBV) exposure may provide protection against detriments in reaction time.
2. Intervention activities that include a gaze stabilization exercise component maintained the slowest reaction speeds after 1 hour of WBV exposure, whereas sitting and walking activities resulted in a further slowing of the slowest reaction speeds.
3. It may be possible for machinery operators to incorporate gaze stabilization activities in occupational environments, either in or out-of-cab, but further evaluation for feasibility and practicality of in-field adoption is required.

## 1. Introduction

Occupational whole-body vibration (WBV) exposure is an unavoidable consequence of operating machinery in various industrial workplaces such as mining [1–3], construction [4], public transportation [5], trucking [6, 7], and agriculture [8], where the likelihood of over-exposure to WBV is high. Related health effects of WBV exposure are not fully understood, though it has been shown to negatively affect proprioception, vestibular function, reaction time, stress, sensory and motor response, and musculoskeletal (MSK) health [9–12]. Postural deficits caused by exposure to vibration have been shown to last for several hours in some individuals [13]. These deficits can lead to a high risk of near-miss incidents (slips, trips, and falls), especially during machine egress [14], and are related to on-farm injuries and fatalities [15].

WBV exposure as a result of prolonged farm machinery operation exposes users to long-term MSK disorders relating to hip and low back pain (LBP), vestibular and balance deficits and short-term cognitive impairments. Individuals exposed to WBV while seated have shown an increased latency period in the neuromuscular reflex loop [16], which controls spinal stability through passive non-contractile tissues, and active muscle recruitment [17]. The reflex loop affects reflex gain and modulates muscle activity in response to a given perturbation [16]. Delayed reflex response and muscle activation due to WBV perturbations may put additional stress on the passive non-contractile tissues leading to ligamentous or vertebral injury. Sitting and its associated postures, such as slouching, combined with WBV exposure can compound associated negative health effects. Flexed lumbar posture experienced while sitting predisposes intervertebral disks to injury through decreased resistance to buckling moments which is magnified by WBV, increasing the probability of low back disorders [18]. Sudden impacts, imposed by WBV or occupational slips, trips and falls combined with a latent reflex loop can overly strain passive supports, ligament and connective tissue in the lumbar spine leading to lumbar pathologies in acute and chronic timeframes [16].

Recent approaches developed to prevent or minimize WBV effects have focused on engineering controls. Seat, steering wheel and suspension systems have been explored, but have proved insufficient in mitigating vibration-related effects in both industrial and personal vehicles in off-road environments [4, 6]. To our knowledge, the implementation of rest protocol or physical activity interventions has not yet been explored as an alternative to engineering controls in an agricultural setting. Previous laboratory studies have shown that a two-minute break following 45 min of WBV exposure is sufficient to wash out any latent reflex response of lumbar and spinal erector muscles [16], supporting the use of short-duration rest break interventions to mitigate the adverse effects of occupational WBV exposure.

With current agricultural practices, it is not possible to eliminate exposure to occupational WBV, but it may be possible to break up extended periods of sitting WBV exposure through a series of short rest breaks throughout the day. Recent work evaluating commercial truck drivers [7] and construction equipment operators [10] has shown that a rest break incorporating upper and lower body stretches during the working day is effective in reducing the negative effects of WBV exposure on reaction time and postural sway. Ouillier et al. [4] report on an exercise-based intervention with a sensorimotor re-stabilization procedure following occupational WBV exposure from upright operation of mining equipment. Their three-minute intervention of a series of trunk exercises after 2 h of standing WBV exposure reduced WBV-induced postural alterations and aligns with our goal of a short intervention that minimizes adverse health effects created by seated WBV. Additionally, gaze stabilization exercise (GSE) has been used to facilitate rehabilitation of vestibular hypofunction linked to balance deficits [19]. Utilizing GSE in a post-WBV intervention activity may provide an additional avenue to alleviate vestibular effects caused by exposure to occupational WBV. GSE offers a time-effective, straightforward intervention that can be performed on the ground or in-cab, regardless of physical ability.

Laboratory-based investigation into occupational WBV is a prerequisite to providing baseline parameters for field-based intervention studies and allows for high fidelity replication of WBV exposure in combination with specifically controlled interventions to elucidate somatosensory effects of occupational WBV. While lab-based investigations cannot expose participants to real-world duration of WBV [8], it can provide a snapshot of what those effects may be and provide guidance in methodological decision-making prior to in-field settings.

The objective of this study is to determine if there are feasible and practical intervention activities that can minimize decrements in cognition, proprioception, and musculoskeletal discomfort related to WBV exposure during agricultural machine operation.

## 2. Methods

### 2.1. Participants

Eleven participants, (10 male, 1 female; aged 40 ± 14 years) with a minimum of one year of experience in operating agricultural or heavy machinery were recruited for this study. Exclusion criteria included: a history of work-limiting pain in the 6 months prior, medical conditions or medications that affect balance, and current or previous head injury. All participants were asked to minimize WBV exposure 24 h prior to each testing session. All participants provided written consent and the study was approved by the University of Saskatchewan Research Ethics Board.

### 2.2. Experimental design

**Figure 1.**
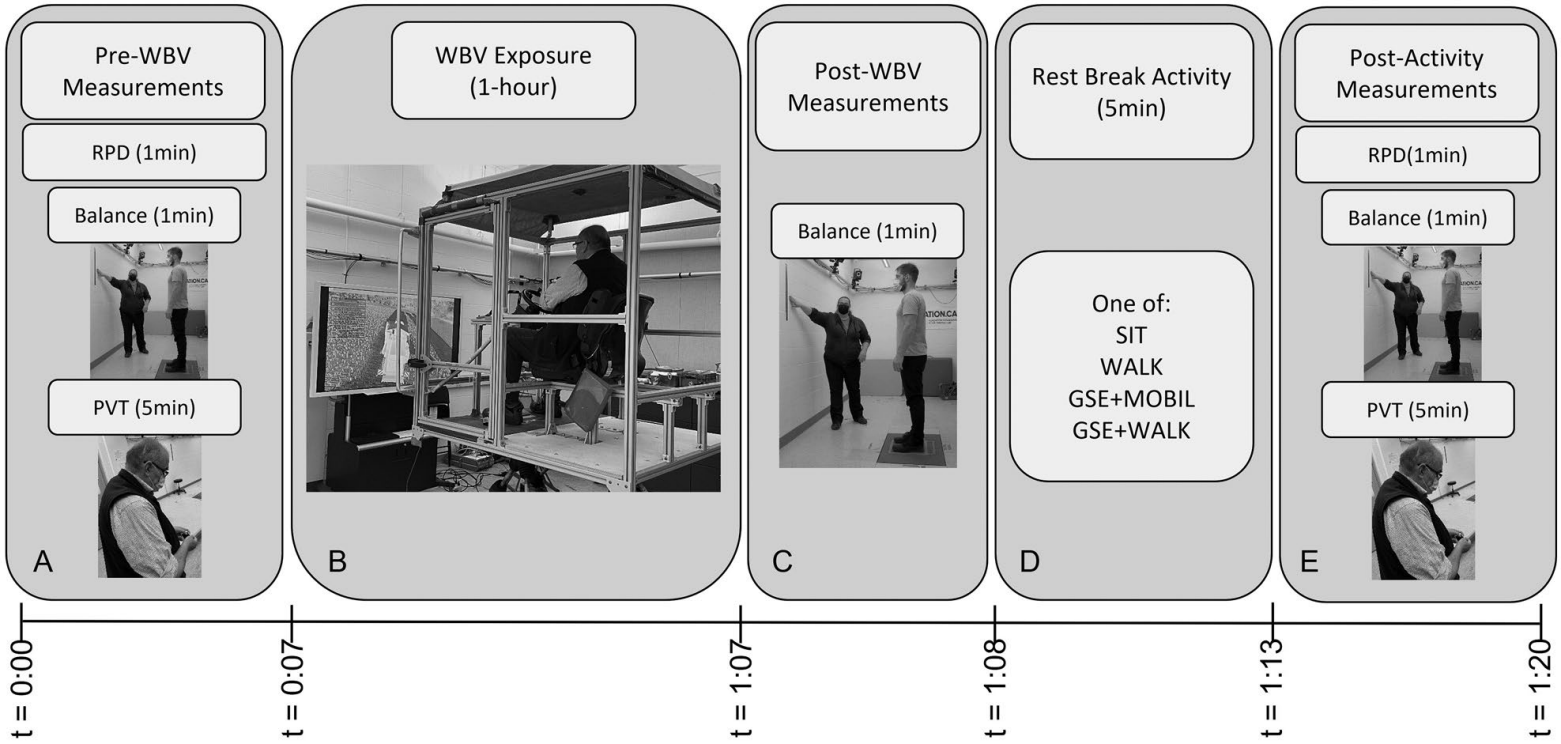
In-lab whole body vibration (WBV) study protocol. Pre-study measurements include a rating of perceived discomfort (RPD), balance and postural sway measurements followed by a 5-minute psychomotor vigilance task (PVT) test (A) Participants were then exposed to 1-hour of WBV (B) Immediately following exposure, balance and postural sway measurements were repeated (C) Participants were then asked to perform one of four selected intervention activities: sitting for 5 min, walking for 5 min, gaze stabilization exercise (GSE) for 2 min and trunk mobility exercises for 3 min (GSE + MOBIL) or GSE for 2 min and walking for 3 min (GSE + WALK) (D). after the 5-minute intervention, post-intervention measurements included RPD, balance and postural sway measurement followed by a 5-minute PVT test (E) for a total test time of 1 h, 20 min. The session timeline is represented at the base of figure.

To investigate the effect of rest break activities immediately following WBV exposure on cognition, proprioception, and musculoskeletal discomfort, we used a repeated measures experimental design. Participants took part in four data collection sessions a minimum of seven days apart. Each data collection session consisted of 1-hour of WBV exposure followed by one of four randomly selected intervention activities, each 5-minutes in duration. Intervention activities included: sitting (SIT); walking (WALK); 2-minutes of gaze stabilization exercise (GSE) coupled with 3 min of trunk mobility exercise (GSE + MOBIL); or 2-min of GSE coupled with a 3-minute walk (GSE + WALK). Rest break activities were each assigned a number from 1 to 4, then randomly assigned *via* a balanced random number generator for each participant. Prior to WBV exposure and following the intervention activity, participants were asked to perform a battery of test measures based on previous studies evaluating the health effects of WBV exposure [4, 20–22]. Measurements were performed in a consistent order as quickly as possible to minimize possible residual or wash-out effects between measures. The test battery prior to WBV exposure included: i) rating of perceived discomfort using Borg’s CR 10 scale [23], ii) balance and postural sway evaluation, and iii) psychomotor vigilance task (PVT) [24]. Balance and postural sway measurement was repeated immediately following WBV exposure. Participants then performed one of four randomly selected intervention activities, followed by the test battery in order: i) postural sway and balance evaluation, ii) PVT, and iii) rating of perceived discomfort using Borg’s CR 10 scale. At the beginning of each testing session participants were given the opportunity to practice test battery measures (Figure 1).

### 2.3. Vibration conditions

All WBV exposures were performed with a R3000 Parallel Robotic System (Mikrolar Inc, Hampton, NH) or ‘rotopod’ fitted with a constructed cab to reflect the dimensions of a mid-sized agricultural tractor based on a New Holland T6 series (CNH Industrial, London, UK) as the basis of our laboratory-based model. A 1-hour WBV exposure was a simulation based on seat-pan accelerations previously collected from farm machinery on farms in the Canadian province of Saskatchewan [8] and exposed participants to the same total energy as the 8-hour standardized exposure action value (1.15 ms^−2^) according to ISO standard 2631-1 [25]. Multiple 4-minute samples were extracted from these acceleration data and imported to vibration analysis software (Vibration Analysis ToolSet, NexGen Ergonomics, Pointe Claire, QC) to construct a simulation signal. A vibration profile of frequency-weighted root-mean-squared (RMS) acceleration of 0.41 ms^−2^ with a dominant median frequency of 4.0 Hz along the vertical axis of the cab was used to simulate in-field WBV exposure [8]. This acceleration profile was double-integrated to displacement, then input as a control program for the rotopod platform. Accelerations at the model cab seat, with a human occupant, were determined with a 6 g tri-axial accelerometer (SXT V1, Biometrics Ltd, Newport UK) embedded in a rubber seat pad and processed according to ISO standard 2631-1^25^ to ensure that the control program was within 5% of the field measurements [8, 14]. While exposed to WBV, participants were asked to play a farming simulation game (Farming Simulator 19, GIANTS Software GmbH, Zurich, Switzerland) to simulate real-world attentiveness and postures during experimental sessions. Upon completion of the WBV exposure, participants exited the vibration simulator and egress direction was documented.

### 2.4. Intervention activities

Immediately following each WBV exposure and simulator egress, participants were asked to perform a balance and postural sway measurement, then perform one of four 5-minute activity interventions:

1. sitting in a chair while engaging in conversation (SIT),
2. walking through hallways adjacent to laboratory (WALK),
3. 2 minutes of gaze stabilization exercises (GSE) followed by 3 minutes of trunk mobility exercises (GSE + MOBIL), or
4. 2 minutes of GSE followed by a 3-minute walk (GSE + WALK).

These intervention activities were chosen either because they are activities that machinery operators may typically perform immediately following machine egress during normal operation (sitting, walking, or trunk mobility exercises), or provide a feasible alternative activity that may be adapted for performance within the machinery cab (GSE) and could be evaluated in future planned in-field tests.

#### 2.4.1. Sitting

For the sitting (SIT) activity, participants were asked to sit in a non-wheeled chair for 5 min while engaging in casual conversation with research staff. Given the potential social nature of breaks from WBV exposure in-field, we decided to engage participants in conversation during this period to simulate in-field situations as realistically as possible.

#### 2.4.2. Walking

For the walking (WALK) activity, participants were asked to walk for 5 min at a self-selected pace in a chosen direction through the hallways adjacent to the laboratory. Participants were advised when 4 min had elapsed in order to ensure that balance and postural sway measurements could be performed as soon as possible after the 5 min of elapsed time.

#### 2.4.3. Gaze stabilization exercise

**Figure 2.**
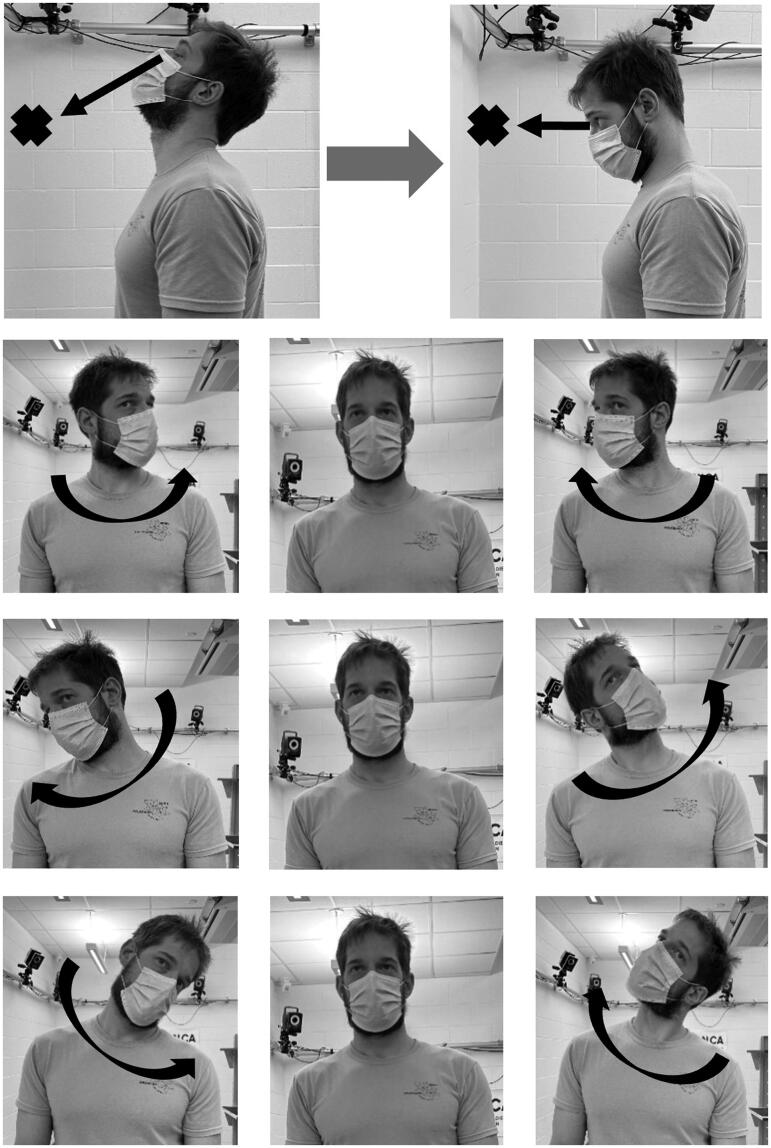
Gaze stabilization exercise (GSE), where participants were asked to focus on a marked point at eye-level approximately 1.5 m distance, while moving their head and neck in cervical flexion and extension, left and right rotation, and diagonally from left shoulder to upper right and right shoulder to upper left, switching movements every 4 repetitions at a comfortable pace.

Gaze stabilization exercise (GSE) consisted of static standing and dynamic head movement with eyes focused on a single point, known as vestibulo-ocular reflex (VOR) exercises [19]. Participants were instructed to maintain eye contact with a single point placed 1.5 m directly in front of them while they moved only their heads into cervical flexion and extension, left and right rotation, and diagonally from left shoulder to upper right and right shoulder to upper left, switching movements every 4 repetitions (Figure 2). Participants were guided by research staff for 2 min of GSE activities. Participants were instructed to keep movement ranges within comfortable limits, while self-selecting a pace that would allow them to maintain eye contact with the single point. GSE has no specific distance to a visual target, although arm’s length distance or across a room is often used [19], as the goal is to maintain visual contact with a specific spot while moving the head.

#### 2.4.4. Trunk mobility exercises

The trunk mobility exercise included trunk flexion and extension, side flexion to the left and right, and twisting left and right while maintaining feet planted at approximately shoulder-width apart. The participants were instructed to only stretch into ranges where they did not experience over a 4/10 on a subjective discomfort scale and to maintain the stretch position for 10 to 15 s. Research personnel verbally guided participants through each motion and cycled through stretching activities for 3 min.

### 2.5. Test measures

This battery of tests was selected for the following reasons. Firstly, previous works evaluating the health effects of WBV exposure [4, 21, 22] have indicated that these outcomes may be sufficiently sensitive to the effects of simulated in-lab vibration levels. Secondly, test battery data collection time is relatively quick (∼7min). Thirdly, data collection techniques can be easily transferred to planned in-field testing situations with same or similar portable measurement devices.

#### 2.5.1. Rating of perceived discomfort

Participants verbally reported their level of musculoskeletal discomfort of 10 body locations using Borg’s category-ratio scale [23]. Body locations included: head and neck, both shoulders, both arms, upper back, lower back, both hips and thighs, both knees, both calves, both feet and ankles. The 11-point scale ranged from a minimum of ‘0′ for no discomfort to ‘10′ for a maximum amount of discomfort. Scores for each location were summed for a possible total score of 100.

#### 2.5.2. Postural sway and balance

All balance and postural sway outcomes were measured using an in-floor mounted force plate (Bertec 6090, Columbus, OH). Center of pressure (CoP) data were collected with Vicon software (Vicon Nexus v2.12.1, Oxford, UK). Participants were asked to maintain an upright standing position at the center of the force plate with arms at their sides for 20 s with eyes open; they were then asked to take 3 to 4 steps in-place to provide an analytical separation of values, then repeated the standing position for another 20 s with eyes closed.

All force plate data were collected at a sample rate of 1000 Hz and processed using a custom algorithm (MATLAB v9.10.0 (R2021a), Natick, MA, The MathWorks Inc). Data were passed through a 4^th^ order, 10 Hz, low-pass Butterworth filter [26–28] and detrended prior to calculating any balance outcomes. The middle 5s from each 20s collection interval was identified and isolated to calculate the following balance measures at each condition (eyes open or eyes closed): root mean square (RMS) displacement (mm) and mean velocity (MeanV) (mm/s) of CoP, and ellipse area (mm^2^) enclosing approximately 95% of the points on the CoP path (ELLIPSE) [28]. Resultant vector distances, which includes both the anterior-posterior and medial-lateral directions [28], are reported for RMS and MeanV outcomes.

#### 2.5.3. Psychomotor vigilance task

The Psychomotor vigilance task (PVT) is typically a standardized 10-minute test that has been shown to be correlated with sleep disruption, cognitive impairments, and mental fatigue [20, 29]. In this study, we used NASA-PVT+ (NASA Psychomotor Vigilance Task Plus for iOS, v1.2.0, Moffett Field CA, NASA) [24], a 5-min reaction time test previously used for measuring performance due to fatigue in laboratory and field studies with comparable reliability as the standard 10 min PVT test [24]. This 5-minute test, as opposed to the more widely used standardized 10 min PVT-192 [30], was used to reduce total test battery time. This specific test was chosen as it is undertaken with a widely used pocket-sized mobile device (Apple iPod or iPhone) which can be easily transported and used in-field. Although there are many versions of mobile-based PVT tests [24, 31, 32], NASA-PVT is open-source and provides latency calibration values for a variety of devices and Apple operating systems, which will allow for widespread self-administration and data collection in planned future studies evaluating on-farm WBV exposure. The PVT has not demonstrated any significant learning curve [24].

To complete this test, participants were directed to sit in a non-wheeled chair with feet planted on the ground. The reaction time test was administered using an iPod (iPod Touch (7^th^ Gen), iOS 15.1, Apple Inc), where participants were asked to react to a visual stimulus on the iPod screen by tapping the screen as quickly as possible using only the thumb of their dominant hand.

Performance outcomes included mean reaction time (MeanRT), inverse mean reaction time, or reaction speed, (InvRT), number of performance lapses (#RTLapse), fastest 10% inverse reaction times (10% FastRT) and slowest 10% inverse reaction times (10% SlowRT). A performance lapse was considered as a reaction time exceeding 500 ms, or the participant touching the screen before prompting with either the dominant or non-dominant thumb. To determine 10%FastRT and 10%SlowRT, inverse reaction times were calculated, ranked, and then the means of the fastest 10% of collected reaction times (fastest 4 to 6 data points depending on number of reactions collected per test), and the slowest 10% of reaction times (slowest 4 to 6 data points depending on number of reactions collected per test) were determined. These two values represent the mean of the fastest (optimal) and slowest (lapsed) reaction speeds within the PVT test.

### 2.6. Analysis

All force plate data was reduced, processed, and completed using a custom algorithm (MATLAB v9.10.0 (R2021a), Natick, MA, The MathWorks Inc), while RPD and PVT data were processed and completed using Microsoft Excel (Microsoft Corporation). For each variable, we evaluated normality using Q-Q plots and Shapiro-Wilk tests. To ensure that the effect of WBV exposure on balance outcomes was consistent between activities, we used a paired t-test to evaluate differences between baseline and post-WBV exposure postural sway outcomes. As there were no differences in any balance outcomes as a result of WBV exposure, and to maintain consistency in comparisons between balance, reaction time, and RPD outcomes, baseline and post-activity data were used to evaluate the effect of each activity on balance outcomes. To determine whether there was an effect of rest break activity on physical, cognitive, and sensorimotor systems, baseline and post-activity data were submitted to a paired t-test in the case of parametric data, or a Wilcoxon rank test for non-parametric and ordinal data. We also report Cohen’s *d* for repeated measures (*d_RM_*) of all parametric outcomes [33], and matched-pairs rank biserial correlation (*r*) for all non-parametric outcomes [34], to report effect size and aid in calculating sample size for planned future in-lab and in-field studies.

To determine whether there were differences across rest break activities, each post-activity data set was normalized to baseline values (reported as % of baseline) for each measure and was entered as the dependent variable in a repeated-measures analysis of variance with Bonferroni correction. An alpha level of *p* < 0.05 was considered statistically significant. In the case of ordinal data (RPD and #PerfLapse for PVT outcomes), a preliminary Friedman’s test was performed on the baseline values. As there was no statistical difference between baseline values, post-activity values were submitted to Friedman’s test and subsequent Wilcoxon rank tests. All statistical tests were carried out using SPSS (SPSS 28, Armonk, NY, IBM Corp).

## 3. Results

### 3.1. Effect of WBV and intervention activity on cognition

**Figure 3.**
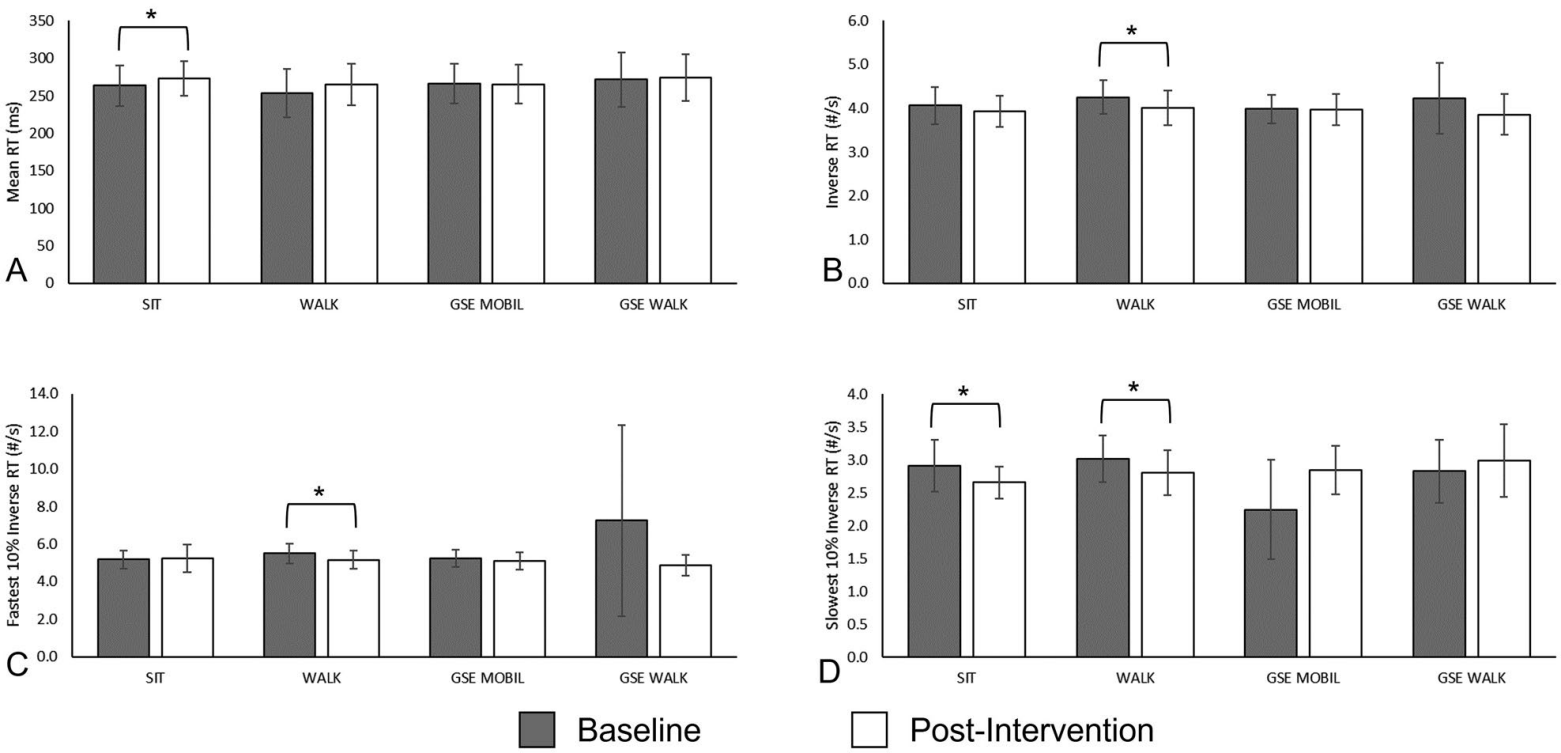
Paired t-test results of cognitive outcomes between baseline and post-activity for each reaction time (RT) outcome: Mean RT (a), inverse RT (B), fastest 10% inverse RT (C), and slowest 10% inverse RT (D) across each activity: SIT, WALK, GSE + MOBIL, and GSE + WALK. Error bars represent 95% confidence intervals of the mean. Significant differences between baseline and post-activity outcomes are noted with (*).

Differences between baseline and post-activity reaction times were observed across several PVT outcomes and various activities. MeanRT was higher by approximately 4% (*p* < 0.05, Cohen’s *d_RM_* =–0.78) after the SIT activity, representing an unfavorable effect, whereas there were no differences between baseline and post-activity MeanRT for any other activity (Figure 3(A), Table 1). After WALK, InvRT (reaction speed) was 5.6% slower than baseline (*p* = 0.005, Cohen’s *d_RM_=*1.07) and 10%FastRT was 6.5% (*p* < 0.05, Cohen’s *d_RM_ =*0.76) slower than baseline, representing unfavorable effects in each RT outcome. Although reaction speed (InvRT) was 8.7% slower than baseline and 10%FastRT was 33.2% slower than baseline after GSE + WALK, these differences did not reach statistical significance. No differences in InvRT or 10%FastRT were observed after any other activity. (Figure 3 (B and C), Table 1).

**Table 1.** Mean differences between baseline and post-activity reaction time, musculoskeletal discomfort, and balance and postural sway outcomes. A sample size of *n* = 11 for all outcome variables. For any significant differences, represented in bold, the direction of the test outcome on operator’s health (favorable effect or unfavourable effect) was also noted.

Outcome	Activity	Baseline	Post	Mean difference (%)	95% Confidence Interval of the mean difference		*p*-value	Direction of health effect	Cohen’s *d*
		Mean ± SD	Mean ± SD		Lower Limit	Upper Limit			
MeanRT (ms)	**SIT**	**263.2 ± 40.3**	**273.0 ± 34.4**	**−9.8 (-3.7%)**	**−18.4**	**−1.3**	**0.028**	**Unfavorable**	**−0.78**
	WALK	253.1 ± 47.7	264.7 ± 41.4	−11.7 (-4.6%)	−26.2	2.9	0.104		−0.54
	GSE + MOBIL	266.1 ± 34.4	265.3 ± 38.3	0.8 (0.3%)	−11.1	12.8	0.881		0.05
	GSE + WALK	271.4 ± 53.6	274.1 ± 46.7	−2.6 (1.0%)	−28.2	23	0.825		−0.07
InvRT (#/s)	SIT	4.06 ± 0.63	3.93 ± 0.53	0.14 (3.2%)	−0.01	0.27	0.053		0.66
	**WALK**	**4.25 ± 0.58**	**4.01 ± 0.58**	**0.24 (5.6%)**	**0.09**	**0.39**	**0.005**	**Unfavorable**	**1.07**
	GSE + MOBIL	3.98 ± 0.49	3.96 ± 0.53	0.02 (0.5%)	−0.06	0.1	0.599		0.16
	GSE + WALK	4.23 ± 1.19	3.86 ± 0.9	0.37 (8.7%)	−0.27	1.01	0.226		0.39
#Lapse (#)*	SIT	1 (0-4)	1 (1-4)				0.730		−0.14
	WALK	2 (1-3)	5 (1-5)				0.073		0.67
	GSE + MOBIL	2 (1-3)	2 (1-3)				0.679		−0.15
	GSE + WALK	1 (0-3)	3 (0-4)				0.305		0.38
10% FastRT (#/s)	SIT	5.17 ± 0.73	5.23 ± 1.11	−0.06 (-1.2%)	−0.53	0.41	0.777		−0.09
	**WALK**	**5.51 ± 0.80**	**5.15 ± 0.72**	**0.35 (6.5%)**	**0.04**	**0.67**	**0.030**	**Unfavorable**	**0.76**
	GSE + MOBIL	5.24 ± 0.71	5.08 ± 0.68	0.16 (3.1%)	−0.7	0.39	0.163		0.45
	GSE + WALK	7.26 ± 7.59	4.85 ± 0.82	2.41 (33.2%)	−2.42	7.24	0.292		0.33
10% SlowRT (#/s)	**SIT**	**2.91 ± 0.59**	**2.66 ± 0.36**	**0.25 (8.6%)**	**0.05**	**0.46**	**0.021**	**Unfavorable**	**0.82**
	**WALK**	**3.02 ± 0.53**	**2.80 ± 0.51**	**0.22 (7.3%)**	**0.03**	**0.46**	**0.037**	**Unfavorable**	**0.60**
	GSE + MOBIL	2.25 ± 1.12	2.85 ± 0.55	−0.61 (-26.6%)	−1.49	0.28	0.157		−0.46
	GSE + WALK	2.83 ± 0.72	2.99 ± 0.82	−0.16 (-5.6%)	−0.58	0.26	0.423		−0.25
RPD	**SIT**	**2 (0-4)**	**3 (1-7)**				**0.024**	**Unfavorable**	**1.0**
	**WALK**	**2 (0-5)**	**2 (1-6)**				**0.024**	**Unfavorable**	**1.0**
	GSE + MOBIL	1 (0-5)	2 (1-6)				0.131		0.73
	GSE + WALK	2 (0-4)	2 (0-6)				0.102		0.8
RMS - eyes open	SIT	5.40 ± 4.60	4.69 ± 2.31	0.71 (13.1%)	−2.30	3.72	0.612		0.16
	WALK	4.53 ± 1.83	5.37 ± 2.64	−0.83 (-18.3%)	−2.61	0.94	0.320		−0.32
	GSE + MOBIL	5.03 ± 1.72	4.19 +/-1.44	0.84 (16.7%)	−0.11	1.79	0.076		0.60
	**GSE + WALK**	**3.80 ± 0.84**	**6.16 ± 2.44**	**−2.36 (-62.1%)**	**−4.02**	**−0.70**	**0.010**	**Unfavorable**	**−0.96**
RMS - eyes closed	SIT	5.99 ± 1.79	5.50 +/-1.79	0.48 (8.0%)	−0.74	1.71	0.402		0.26
	WALK	5.88 +/-2.92	6.98 +/-3.65	−1.10 (-18.7%)	−2.90	0.68	0.202		−0.41
	GSE + MOBIL	5.76 +/-2.18	5.83 +/-3.18	−0.07 (-1.2%)	−2.30	2.15	0.943		−0.02
	GSE + WALK	6.71 ± 2.79	5.92 +/-4.04	0.79 (11.8%)	−2.56	4.13	0.612		0.16
Meanv - Eyes Open	SIT	11.12 ± 2.26	10.40 ± 2.93	0.72 (6.5%)	−1.48	2.93	0.482		0.22
	WALK	10.70 ± 2.51	11.74 +/-3.91	−1.04 (-9.7%)	−3.67	1.6	0.401		−0.26
	**GSE + MOBIL**	**11.48 +/-4.38**	**9.26 ± 2.03**	**2.22 (19.3%)**	**0.24**	**4.2**	**0.031**	**Favorable**	**0.75**
	GSE + WALK	10.53 ± 3.64	10.40 ± 2.93	0.12 (1.1%)	−1.66	1.91	0.880		0.05
Meanv - Eyes Closed	SIT	19.39 ± 13.94	15.16 ± 7.02	4.22 (21.8%)	−1.25	9.7	0.117		0.52
	WALK	16.01 +/-6.46	16.17 ± 6.89	−0.15 (-0.1%)	−5.11	4.8	0.947		−0.21
	GSE + MOBIL	16.37 +/-6.66	15.33 +/-7.19	1.05 (6.4%)	−3.55	5.64	0.624		0.15
	GSE + WALK	17.36 ± 7.50	15.83 +/-11.52	1.53 (8.8%)	−2.80	5.86	0.450		0.24
Ellipse - eyes open	SIT	53.76 ± 66.00	53.45 ± 43.13	0.31 (0.6%)	−42.56	43.18	0.987		0.01
	WALK	38.17 +/-22.61	58.16 +/-63.75	−19.99 (-52.3%)	−64.17	24.18	0.337		−0.30
	GSE + MOBIL	41.32 +/-28.50	37.94 +/-19.72	3.38 (8.2%)	−16.53	23.29	0.713		0.11
	GSE + WALK	29.98 +/-12.04	59.16 +/-51.67	−29.19 (-97.3%)	−61.28	2.9	0.070		−0.61
Ellipse - eyes closed	SIT	59.61 ± 40.47	55.22 ± 41.62	4.39 (7.4%)	−8.99	17.78	0.481		0.22
	WALK	85.64 ± 108.35	63.05 ± 43.54	22.58 (26.4%)	−36.23	81.4	0.412		0.26
	GSE + MOBIL	73.41 ± 66.06	63.89 ± 61.00	9.52 (13.0%)	−45.21	64.25	0.706		0.12
	GSE + WALK	88.04 ± 64.28	76.39 ± 79.18	11.65 (13.2%)	−53.13	76.43	0.697		0.12

Unfavorable differences between baseline and post-activity in 10%SlowRT were observed following both SIT (8.6% slower, *p* = 0.021, Cohen’s *d_RM_* =0.82) and WALK (7.3% slower, *p* = 0.037, Cohen’s *d_RM_* =0.60), but not in either activity incorporating GSE: GSE + WALK or GSE + MOBIL. Although both GSE + MOBIL and GSE + WALK presented an increase in 10%SlowRT, the differences between baseline and post-activity did not achieve significance (Figure 3(D), Table 1).

### 3.2. Effect of WBV and activity on musculoskeletal discomfort (RPD)

There was a trend for higher total RPD outcomes across activities, with significantly higher total RPD scores observed post-activity with SIT and WALK (*p* < 0.05, *r* = 1.0), both representing an unfavorable effect on operator health. We report no significant differences between baseline and post-activity RPD scores for GSE + MOBIL or GSE + WALK (Table 1).

### 3.3. Effect of WBV and activity on postural balance and sway

We observed differences between baseline and post-activity RMS values with eyes open in participants after GSE + WALK (62% higher, *p* = 0.01, Cohen’s *d_RM_* =-0.96), and in sway MeanV with eyes open in participants after GSE + MOBIL (19.3% slower, *p* = 0.031l, Cohen’s *d_RM_* =0.75). There were no other differences between baseline and post-activity balance or postural sway outcomes, with either eyes open or closed, or after any other activities. Generally, balance and postural sway measurements were highly variable in this sample, and there were no discernable patterns in balance or postural sway measurements across any activities (Table 1).

### 3.4. Effects across activities on reaction time

**Figure 4.**
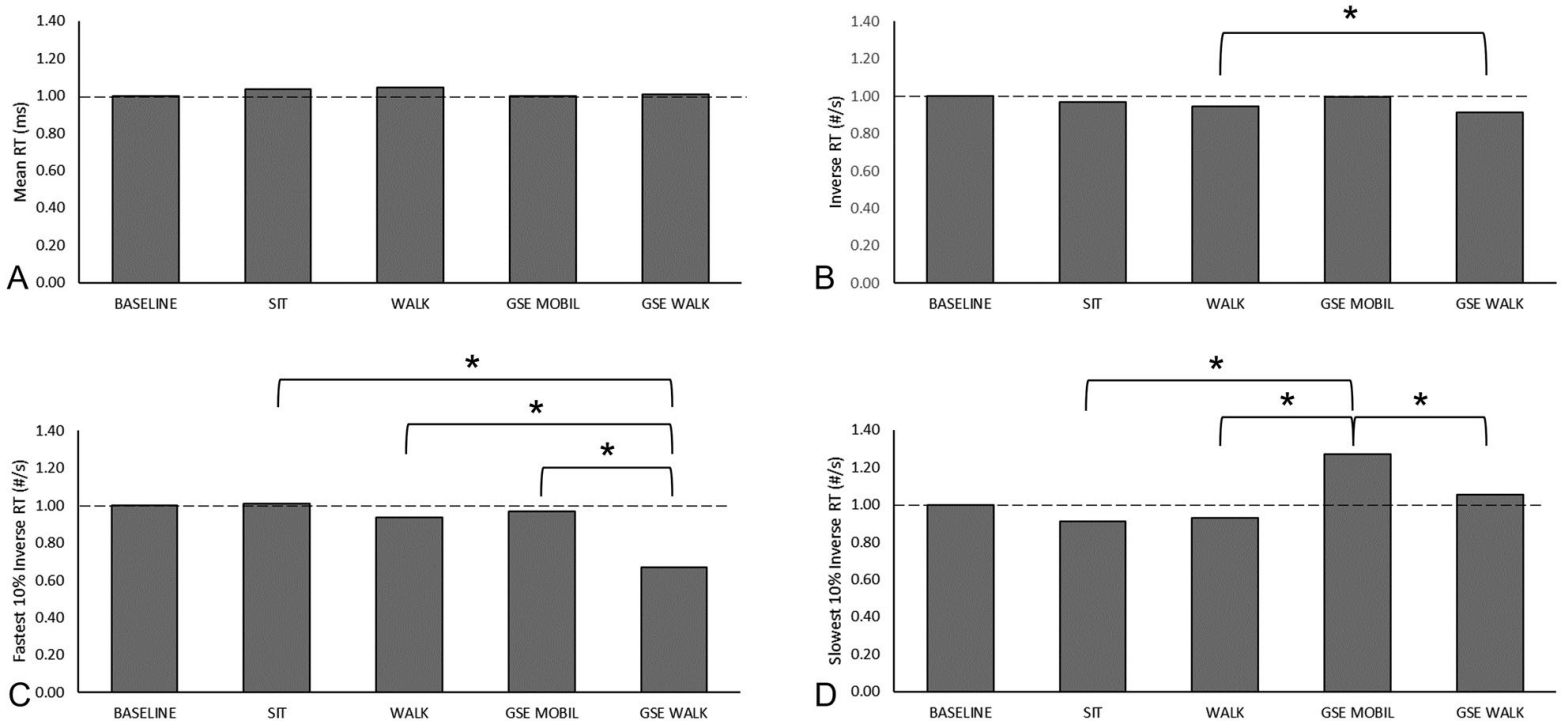
Repeated measures ANOVA results of normalized post-activity cognitive outcomes between all activities: SIT, WALK, GSE + MOBIL and GSE + WALK. Reaction time (RT) outcomes include mean RT (a), inverse RT (B), fastest 10% inverse RT(C), and slowest 10% inverse RT (D). Significant differences in normalized post-activity outcomes are noted with (*). normalized baseline values are included for comparison.

We observed differences in normalized post-activity reaction time outcomes between various activities. No differences in normalized MeanRT were observed between activities (Figure 4-A), but normalized InvRT between WALK and GSE + MOBIL activities were significantly different (*p* = 0.005). Although there were no significant differences in InvRT between GSE + MOBIL and the other two activities (SIT and GSE + WALK), there is an observable trend where GSE + MOBIL displays a reaction time response that is very similar to baseline, but all other evaluated activities produced slower reaction time responses than baseline (Figure 4-B).

Differences in normalized 10%FastRT and 10%SlowRT were also observed across various activities. Normalized 10%FastRT after GSE + WALK was slower than normalized 10%FastRT after SIT (*p* < 0.001), WALK (*p* < 0.001) and GSE + MOBIL (*p* < 0.001). Each of normalized 10%FastRT outcomes after SIT, WALK, and GSE + MOBIL are very close to the baseline value of 1.0 and were able to maintain baseline reaction time, whereas after GSE + WALK, 10%FastRT slowed down to 67% of baseline, leading to a slower response time. No differences were observed between other activities (Figure 4-C). Normalized 10%SlowRT after GSE + MOBIL was higher than normalized 10%SlowRT after each of SIT (*p* < 0.001), WALK (*p* < 0.001), and GSE + WALK (*p* = 0.015). Additionally, normalized 10%SlowRT after GSE + MOBIL was 27% higher than baseline 10%SlowRT (Figure 4-D).

### 3.5. Effects across activities on proprioception and musculoskeletal discomfort

**Figure 5.**
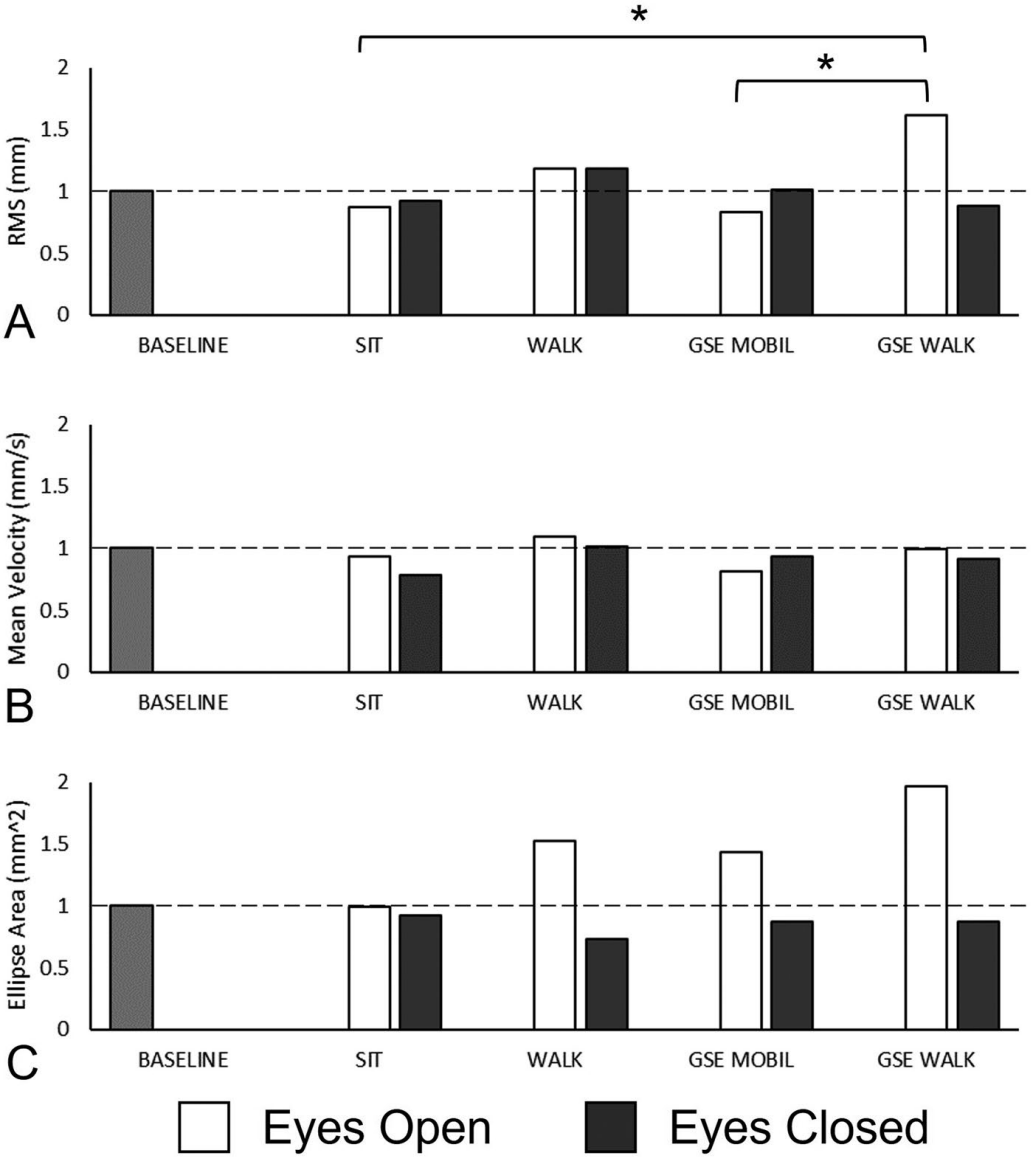
Repeated measures ANOVA results of normalized post-activity proprioceptive outcomes between all activities: SIT, WALK, GSE + MOBIL and GSE + WALK. Balance and postural sway outcomes, for both eyes open and eyes closed conditions, include: root mean square of postural sway (RMS), mean velocity of postural sway, and ellipse area. Significant differences in normalized post-activity outcomes are noted with (*). normalized baseline values are included for comparison.

Few differences in balance and postural sway outcomes were observed between activities. Differences in normalized RMS – Eyes Open were observed between GSE + WALK and SIT (*p* = 0.024) and GSE + WALK and GSE + MOBIL (*p* = 0.016), where normalized RMS was higher after the GSE + WALK in each case (Figure 5(A)). There were no other differences in any other balance and postural sway measurements between any activities. There were no differences in RPD between activities (Figure 5(B and C)).

## 4. Discussion

This study has identified that occupational low-frequency WBV exposure has a negative effect on cognitive function as measured through reaction time testing. Furthermore, we report that interventions incorporating GSE can provide some protection against decrements in reaction time. This is especially important when related to 10%SlowRT outcomes, as this outcome represents the slowest 10% of tested reaction times, or possible worst-case scenario. While WALK and SIT activities showed slower 10%SlowRT outcomes post-activity than baseline, 7.3% and 8.6% respectively, outcomes trended toward maintaining reaction time when activities included GSE combined with either mobility exercises or walking. Essentially, after walking or sitting, the slowest RTs were slower, whereas there were no changes between baseline and post-activity slowest RTs after an activity that incorporated GSE. This outcome is of particular interest as it identifies that of the tested activities, sitting and walking for five minutes after WBV exposure can lead to the slowest reaction times becoming even slower, potentially exacerbating a worst-case scenario for machinery operators. Conversely, after activities incorporating GSE, although we did not observe an improvement in reaction times, maintenance of these slowest reaction times was observed, which may not improve on the worst-case scenario related to reaction time but did not degrade the worst-case scenario. Although we have identified some effects related to balance and musculoskeletal discomfort, these outcomes were highly variable across participants and activities.

The current work provides certain insight regarding specific rest break activities that may mitigate the negative decrements of WBV exposure on cognition, namely reaction time. When GSE was paired with any other activity, either walking or trunk mobility exercises, notable differences in reaction time were observed. In comparisons of baseline and post-activity reaction time outcomes, GSE + MOBIL and GSE + WALK were the only rest break activities that did not demonstrate significant decrements in reaction time across any reaction time outcome. Of note, both SIT and WALK activities resulted in 7% to 8% slower responses in 10%Slow RT outcomes, situating that the slowest responses were even slower after each of these activities. Although not reaching a level of significance, GSE + MOBIL and GSE + WALK activities presented 10%SlowRT outcomes that were greater (faster) than baseline values, which may present a trend in improvement in the slowest reaction times. Across activities, normalized reaction time outcomes provide similar results. Normalized 10%SlowRT after GSE + MOBIL was higher after each other evaluated activity, showing that GSE + MOBIL may be a promising activity to aid in maintaining reaction time speed. The selected GSE exercises were developed based in vestibular adaptation, which aims to reduce the symptoms of a provoking stimulus [19, 35, 36], in this case the vestibular and proprioceptive disturbances induced *via* WBV exposure [11, 12]. Applications of GSE include improving gaze stability during head movement in patients with vestibular hypofunction [37, 38], mild cognitive impairment [39], dizziness in elderly patients [36], and postural control [40]. Common to each of these applications, and present in WBV exposure, is the disturbance of the proprioceptive feedback loop, especially in transitional states such as recovery from a shock impact during WBV exposure or removal from WBV exposure immediately before and after cab egress. As cognition and balance are related to one another [41], it may be possible for rest break activities to incorporate a GSE component to mitigate each of these negative health effects resulting from high levels of WBV exposure. Differences in baseline and post-activity reaction times could potentially be due to ocular reset, or alternatively an interaction of vision and the vestibular system related to postural control [39]. Additionally, it is unsure if the observed effect in reaction time is a result of GSE only or other factors, as our chosen activities included a 2-minute GSE activity with 3 min of a dynamic activity to maintain consistency of a 5-minute rest break activity period across all interventions. To our understanding, this is the first study to incorporate GSE-based activities as an intervention after WBV exposure, and the application of GSE, both alone and in combination with other rest break activities is worth further exploration in both controlled laboratory and field environments.

Our results are consistent with findings of similar studies reporting that occupational WBV exposure generally yields decrements in reaction time or cognitive outcomes [20, 42–45]. These effects seem to emerge largely following shorter, lower amplitude exposures at frequency patterns outside occupational WBV exposure patterns of machinery, within the frequency range of 4.1 to 5 Hz [8, 26]. The WBV exposure conditions and reaction time outcomes in the present study, are most aligned with work reporting reaction time increases ranging from 5% [43] to 25% [42], placing our findings of an increase in reaction time of ∼10% after sitting or walking rest break activities within reasonable confidence. This seems to suggest that differing effects on cognitive performance reported in this study are not by random chance, but rather a product of different exposures stimulating certain mechanistic pathways.

Conversely, certain works either report no changes in reaction time, or even improvements in reaction time, after or during WBV exposure [10, 46–48]. Of note is work by Ljunberg and Neely [10] where participants were exposed to various combinations of noise and seated WBV for approximately 45 min then completed a search and memory test pre- and post-WBV exposure. Participants produced faster reaction time responses in post-WBV conditions, but also performed a higher number of errors in reaction time post-WBV, so it could be argued that there was no actual overall improvement in reaction time. Although we report slower reaction time after WBV exposure, we do not report any differences in performance lapses (responses >500ms, participant taps the screen with non-dominant thumb, or participant taps screen when no visual signal appears) between pre- and post-WBV after any rest break activity.

Similar works also report discrepancies in balance and postural sway outcomes following WBV exposure, with certain studies reporting decrements in postural sway after WBV exposure [4, 22, 49] and others reporting no [50] or minimal decrements [20] in postural sway outcomes after WBV exposure. In these cases where balance decrements after WBV exposure were reported, several elements which were not present in the current study may be related to disturbances in balance and postural sway outcomes. For example, Park et al. [49] exposed participants to two 2-hour sessions with a 30-minute break in between, having the participants remain seated for a total of 4.5 h, and report decrements in postural sway mean velocity. Other studies reporting decrements in postural sway outcomes either had participants stand during WBV exposure [4], or were evaluating balance after WBV exposure from a quad bike [20, 22] where participants are required to undertake an active riding stance [51], which involves the rider changing their body position and the centre of gravity to optimize stability. Although the driving terrain and vibration profile were highly controlled in each of these studies, this active riding position—as opposed to a passive seated position in a traditional tractor or vehicle seat—engages lower limb muscles potentially contributing to muscle fatigue and leading to differences in balance and postural sway outcomes even though WBV exposure conditions were highly similar. Overall, WBV exposure levels and duration in our study, in combination with passive sitting, were likely not sufficient to induce detectable perturbations in balance, given the comparatively short vibration period (1 h vs 2 to 4 h). In this case, reaction time may be a more sensitive indicator of performance decrements due to WBV exposure, and we recommend further study, especially in workplace or field environments with longer durations of WBV exposure.

Although the recommended rest break length and activities required to offset the negative health effects of WBV exposure and prolonged sitting are uncertain, it is possible to infer a range of appropriate rest break lengths and activities that are also feasible for practical implementation in-field. In a pilot trial to determine study protocol feasibility, participants completed three PVT tests: before WBV exposure (Pre-WBV), immediately following WBV exposure (Post-WBV), and again after an additional 5-minute period of sitting (Post-Rest). The reaction speed (inverse RT) of this particular trial showed slowing of reaction time immediately following WBV exposure, but no differences between reaction speed (inverse RT) outcomes after sitting for a total of 10 min after WBV exposure (5-min PVT test + sitting for 5 min) (Supplemental material). This suggests that reaction time is likely to return to normal Pre-WBV levels following 10 min of sitting post-exposure. We selected sitting, walking, trunk mobility exercises and GSE as intervention activities for this study as they are easy to perform, easy to instruct and do not require peripheral or extra equipment. These activities also share similarities with other typical activities that agricultural machinery operators may perform during their day-to-day tasks e.g. circle check of equipment, sit and talk with other operators, and simple stretches upon vehicle egress. Additionally, it is possible to perform GSE activities either in or out of the tractor cab, in either a sitting or standing position. It may be that the lack of compliance reported in previous works evaluating the effectiveness of intervention programs to reduce WBV exposure [52] is not due to insufficiencies in related outreach or information distributed to operators and employers in such studies, but instead related to suggested activities that may lack easeful integration into typical working patterns. Further on-farm evaluation of typical operator behaviour, involving both observational and qualitative studies, is necessary to further develop these rest break activities and implementation strategies to ensure feasible on-farm application and adoption.

Although study participant characteristics are representative of typical operators in Western Canadian agricultural workplaces, there are certain characteristics and relationships between age, reaction time, and MSK discomfort worth noting. The average age of this sample was 40yrs ±14 years, with a maximum participant age of 68 years. Older machinery operators are more likely to exhibit signs of MSK disease such as low back pain and osteoarthritis [53], which may also be work-limiting. Throughout this study, and prior to each data collection session, we inquired each participant regarding the incidence of work-limiting injuries within the past 6 months or since their previous data collection session. No work limiting pain or discomfort was reported by any participant prior to any testing session. Additionally, although there was a requirement of a minimum of 1 year of heavy machinery operation, this was for the purposes of ensuring that operators were comfortable in performing machinery ingress and egress to expedite health effect data collection immediately following WBV exposure. Participants also reported no LBP or MSK diseases, such as osteoarthritis. There may be the possibility that RPD outcomes, especially in older participants, may represent the potential aggravation of MSK diseases, but no condition of note was reported. As older operators may be at higher risk of work-limiting LBP or MSK diseases, after cumulative WBV exposure over multiple seasons, we recommend further evaluation in the older population. Also worth considering are potential relationships between participant age and reaction time outcomes, especially while driving or performing driving-simulated activities, where older drivers exhibit slower reaction times [54]. To determine if such relationships were present in this sample, we performed Pearson’s correlation in a *post hoc* analysis between participant age and each post-activity reaction time outcome. A single significant correlation between 10%SlowRT and age after the walking rest break activity, where older participants had slower reaction times, was observed. There were no other significant relationships between age and reaction time outcomes, but there were observable trends where older participants had a tendency to produce slower reaction speeds overall. As a single relationship was observed in this sample, it may be possible that stronger, more notable relationships may exist in a larger, broader sample of machinery operators after WBV exposure, and we recommend further evaluation. Another relationship to consider between health outcomes is the relationship between reaction time and MSK pain or discomfort, where higher MSK pain is associated with reduced reaction time [55]. Through *post hoc* Spearman’s correlation, we explored potential relationships between reaction time and RPD outcomes. Even though there were no significant relationships, there was a trend towards slower reaction times in participants with higher RPD outcomes. This is of importance, as individuals with chronic pain may have a higher incidence of reduced reaction time [56], and may be more susceptible to trips and falls [57]. This is of note in the current work as the most frequent tractor-related injury occurs upon mounting and dismounting [58]. Further targeted studies evaluating the relationship between reaction time, MSK pain and WBV exposure are recommended to further elucidate the relationships between age, reaction time, and MSK pain or discomfort, especially following WBV exposure.

### 4.1. Strengths and limitations

This study has several strengths that contribute to ecological validity: participant characteristics, vibration exposure characteristics, test battery to measure health outcomes, chosen intervention activities, and study novelty. First, we recruited participants who were either actively farming or familiar with mechanized agricultural practices, with a minimum of one year of agricultural equipment use; thus participants were neither vibration- nor equipment-naïve. This eased simulator ingress and egress, which expedited acquisition study health effect data. We also ensured that the study participant’s age and experience range is consistent with those found in farming communities in Western Canada. Second, the applied WBV exposure waveform was generated using ecologically valid vibration waveforms collected from Western Canadian farms [8] to best simulate on-farm vibration exposure. We also exposed participants to the longest possible vibration duration (1-hour) using this waveform ensuring the same total energy as the 8-hour standardized exposure action value (1.15 ms^−2^) according to ISO standard 2631-1 [25]. Third, our health effect test battery, including RPD, balance and postural sway measurements, and PVT for reaction time outcomes, was approximately seven minutes in duration. This short data collection period ensures that any observed effects are more likely to be an uninterrupted result of WBV exposure with the minimal period for wash-out due to extended data collection time post-WBV exposure. This short test battery is also easily adaptable for use with portable devices and can be duplicated in-field in actual occupational exposure environments. Fourth, the selected and tested interventions are practical and feasible for on-farm and in-field use and can be modified to fit operators’ schedules and mobility. Although not tested in the cab, it may be possible to perform GSE both in-cab prior to machine egress and on the ground in combination with dynamic activity, such as trunk mobility exercises or walking, as tested. As the independent effect of GSE on reaction time or balance and postural sway was not evaluated in this work, we recommend further in-lab and in-field evaluation of GSE in relation to mitigating the adverse effects of WBV exposure. Fifth, to our understanding, this is the first application of vestibular rehabilitation techniques, specifically GSE, used as an intervention after WBV exposure. This is also the first study to evaluate a variety of interventions post-WBV and determine the health and performance effects of such interventions. This study complements and expands on previous work evaluating the effect of sensorimotor exercise on balance and postural sway after WBV exposure [4]. This novel work provides the rationale to pursue in-field testing with workplace-representative vibration duration and intensity, which will also allow for practicality and feasibility testing of the chosen intervention activities.

The present study also has certain limitations. First, although the study sample size is large enough to measure a discernable effect in reaction time, there was no observed effect in balance or postural sway. Second, we test a non-exhaustive list of rest break activities in this work. Other activities that may be worthwhile exploring may include upper body stretches, a combination of upper and lower body stretches, etc. There may also be other mobility activities that are already components of typical on-farm or in-field activities or may offer easy integration into such environments. Third, although we focused on including rest break activities with high potential for in-field practicality and feasibility, the actual practical uptake of these interventions in field applications is unknown. Fourth, in this present study, we limit exposure evaluation to WBV only. Evidence from other studies evaluating agricultural or industrial machinery exposures also evaluates lighting [46] and noise [59]. It has been shown that WBV and noise exposures due to machinery operation may result in similar adverse health effects such as cognitive reaction time, muscle reaction time, balance and postural sway, stress, and drowsiness [60–63]. To further discern the contribution of these adverse exposures on health effects and to further identify and develop strategies to mitigate resulting negative health effects, we recommend more extensive work with larger sample sizes and in-field environments.

## Conclusions

The results of this work are threefold. First, we have identified that occupational WBV exposure has a negative effect on cognitive function as measured through reaction time testing. Although there may also be an undetected effect on balance and postural sway, our results show that reaction time is likely a more sensitive measure of the negative health effects resulting from WBV exposure in this controlled environment. Second, we report that a minimum of 5 min rest break activity duration following WBV exposure may have a higher potential to mitigate the negative effects of WBV exposure related to reaction time than passive sitting. Third, we report that a post-WBV exposure activity including both GSE and an active component, such as walking or trunk mobility exercises, appears to provide protection against decrements in reaction time compared to passively sitting for 5 min, specifically related to 10%SlowRT outcomes. In situations where a reduction in worker MSK pain or discomfort is sought, we recommend the adoption of GSE prior to cab egress followed by an activity, such as trunk stretching or walking as evaluated in this work. Although walking may also provide other cognitive and musculoskeletal benefits, as well as providing a break from WBV, for practical reasons it cannot provide a protective advantage prior to machine egress. Thus, we recommend that future research in agricultural and industrial environments investigate the real-world effectiveness of in-cab GSE activities. Further field studies are required to validate these in-laboratory identified activities in a practical and feasible manner.

## Supplementary Material

Supplemental Material

## Data Availability

Data is available upon request by contacting the corresponding author.
