# Supplementary material for: The effect of selected rest break activities on reaction time, balance, and perceived discomfort after one hour of simulated occupational whole-body vibration exposure in healthy adults
Source: Ann Med. 2023 Aug 12;55(2):2244965. doi: 10.1080/07853890.2023.2244965 (PMC10424600; doi:10.1080/07853890.2023.2244965)
Supplement: Supplemental Material [file IANN_A_2244965_SM9926.docx]

Supplemental Material:

**Table S1.** Reaction time outcomes from pilot session where participants completed a 5-minute psychomotor vigilance task (PVT) at baseline prior to 1-hour of whole body vibration (WBV) exposure (Pre-WBV), immediately following WBV exposure (Post-WBV), then again after sitting for an additional 5 minutes (Post-Rest).

| Test Outcome | Time interval | Mean ± SD |
| --- | --- | --- |
| MeanRT (ms) | Pre-WBV | 258.8 ± 35.2 |
|  | Post-WBV | 295.4 ± 46.5 |
|  | Post-Rest | 286.2 ± 44.9 |
|  |  |  |
| InvRT (#/s) | Pre-WBV | 4.15 ± 0.76 |
|  | Post-WBV | 3.75 ± 0.50 |
|  | Post-Rest | 3.67 ± 0.70 |
|  |  |  |
| #Lapse (#)* | Pre-WBV | 4.09 ± 9.43 |
|  | Post-WBV | 6.27 ± 11.60 |
|  | Post-Rest | 8.73 ± 19.26 |
|  |  |  |
| 10% FastRT (#/s) | Pre-WBV | 5.58 ± 1.79 |
|  | Post-WBV | 4.95 ± 0.65 |
|  | Post-Rest | 5.16 ± 0.75 |
|  |  |  |
| 10% SlowRT (#/s) | Pre-WBV | 2.92 ± 0.55 |
|  | Post-WBV | 2.39 ± 0.53 |
|  | Post-Rest | 2.74 ± 0.77 |

**
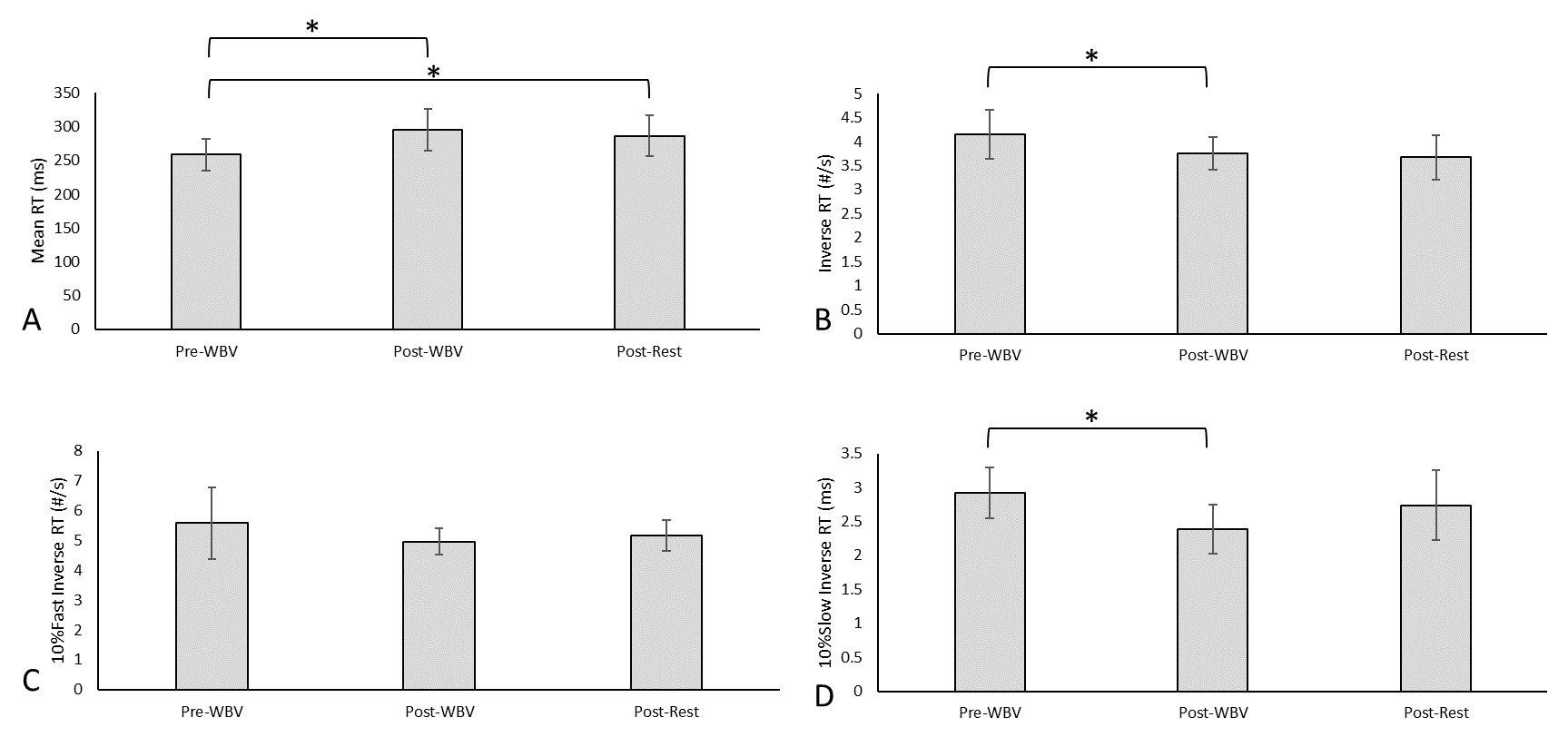
**

**Figure S1.** Paired t-test results of cognitive outcomes between baseline (Pre-WBV), immediately following whole-body vibration (WBV) exposure (Post-WBV), and after sitting for an additional 5 minute period (Post-Rest) for each reaction time (RT) outcome: Mean RT (A), Inverse RT (B), Fastest 10% Inverse RT (C), and Slowest 10% Inverse RT (D). Significant differences between outcomes are noted with (*).
